# Editorial: Reviews in gastrointestinal and hepatic pharmacology: 2024

**DOI:** 10.3389/fphar.2026.1961283

**Published:** 2026-08-26

**Authors:** Anna Maria Giudetti, Ralf Weiskirchen

**Affiliations:** 1 Department of Biological and Environmental Sciences and Technologies, University of Salento, Lecce, Italy; 2 Institute of Molecular Pathobiochemistry, Experimental Gene Therapy and Clinical Chemistry (IFMPEGKC), RWTH Aachen University Hospital, Aachen, Germany

**Keywords:** gastrointestinal disease, liver, low-grade inflammation, MASH, MASLD, microplastics, TGF-β, therapy

Gastrointestinal and liver diseases continue to pose a significant global burden, requiring a deeper understanding of their underlying mechanisms and the development of safer, more effective therapies across the care continuum (Devarbhavi et al., 2023; Gan et al., 2025). In this Research Topic collection, several contributions analyze key molecular pathways in hepatology and oncology. These range from the tumor-suppressive functions of F-box and WD repeat domain-containing 7 (FBXW7) in gastrointestinal cancers to the context-specific effects of transforming growth factor-β (TGF-β) superfamily signaling in liver fibrosis, steatohepatitis, and hepatocellular carcinoma. Additional research highlights both emerging liver toxins, such as microplastic-induced hepatotoxicity, and potential protective interventions, including the hepatoprotective properties of gallic acid and the impact of intravenous magnesium sulfate on cirrhosis outcomes. A significant portion of this issue is dedicated to metabolic dysfunction-associated liver and gastrointestinal diseases, with particular emphasis on metabolic dysfunction-associated steatotic liver disease (MASLD). The roles of mitochondrial homeostasis, Hippo-YAP1/TAZ signaling, cholesterol-associated proteins, and endocrine factors such as fibroblast growth factor 21 (FGF21) are explored in depth. Collectively, these contributions illustrate how altered energy metabolism, lipid handling, and intracellular signaling actively drive hepatic inflammation, fibrosis, and disease progression.

Translational and clinical studies in this issue focus on optimizing pharmacotherapy in digestive medicine. These include a comparison of the effectiveness of biologics and biosimilars in treating inflammatory bowel disease (IBD), evaluations of sedation techniques using ciprofol for elderly patients undergoing endoscopy, studies of vonoprazan-based dual therapy for *Helicobacter pylori*, and investigations into mechanism-based management of opioid-induced constipation using opioid-receptor antagonists. These articles collectively show how combining mechanistic pharmacology with comprehensive clinical assessment can lead to more precise and patient-centered treatment of gastrointestinal and liver disorders.

To provide a conceptual overview of the mechanistic links and therapeutic opportunities discussed across this Research Topic, Figure 1 summarizes the principal drivers and molecular pathways involved in MASLD and its progression. The schematic integrates metabolic and environmental risk factors with key intracellular signaling networks, including TGF-β/Smad and RXR-mediated nuclear receptor pathways, that promote hepatic inflammation, stellate cell activation, and fibrogenesis. In addition, the figure highlights representative pharmacological strategies targeting different aspects of disease pathology, illustrating how advances in molecular understanding are guiding the development of more mechanism-based therapies for gastrointestinal and liver diseases.

**FIGURE 1:**
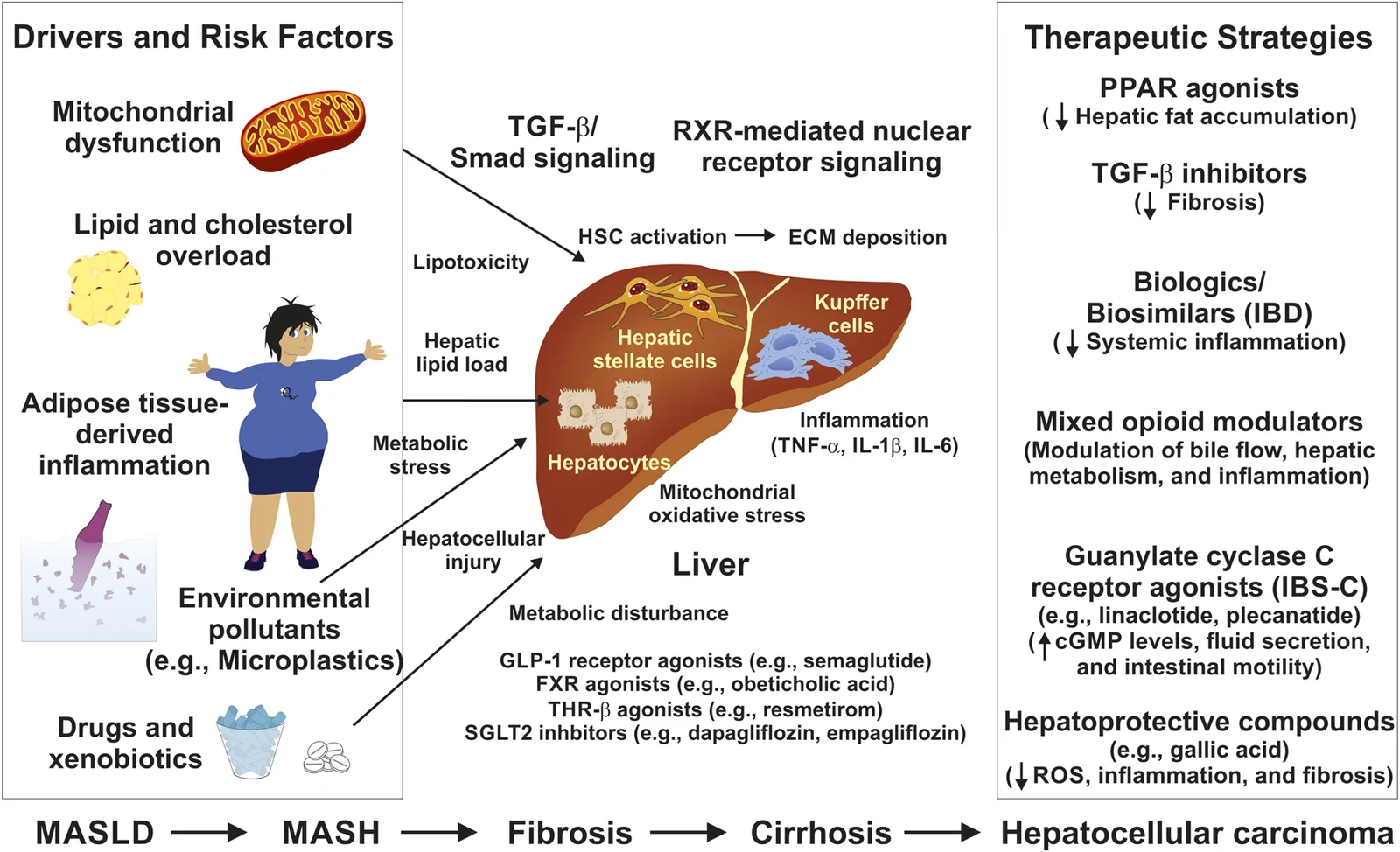
Mechanistic drivers and therapeutic targets in gastrointestinal and liver diseases. The scheme summarizes major risk factors, molecular pathways, and therapeutic strategies involved in the progression of metabolic dysfunction-associated steatotic liver disease (MASLD). Metabolic and environmental drivers, including mitochondrial dysfunction, lipid and cholesterol overload, adipose tissue-derived inflammation, environmental pollutants (e.g., microplastics), and drugs or other xenobiotics, promote hepatic lipid accumulation, lipotoxicity, metabolic stress, and hepatocellular injury. These processes induce mitochondrial oxidative stress and inflammatory responses mediated in part by Kupffer cells releasing cytokines such as TNF-α, IL-1β, and IL-6. In parallel, TGF-β/Smad and RXR-mediated nuclear receptor signaling drive hepatic stellate cell activation and extracellular matrix deposition, leading to fibrosis. Disease progression may advance from MASLD to metabolic dysfunction-associated steatohepatitis (MASH), followed by fibrosis, cirrhosis, and hepatocellular carcinoma (HCC). The right panel highlights selected therapeutic strategies targeting different aspects of disease pathology, including PPAR agonists, TGF-β inhibitors, biologics/biosimilars used in inflammatory bowel disease (IBD) to reduce systemic inflammation, opioid-receptor modulators affecting bile flow and hepatic metabolism, guanylate cyclase C receptor agonists that increase cGMP signaling and intestinal fluid secretion, and hepatoprotective compounds that reduce reactive oxygen species (ROS), inflammation, and fibrosis. Additional pharmacological approaches used in MASLD include glucagon-like peptide-1 (GLP-1) receptor agonists, farnesoid X receptor (FXR) agonists, thyroid hormone receptor-β (THR-β) agonists, and sodium-glucose cotransporter-2 (SGLT2) inhibitors.

Xu et al. reviewed retinoid X receptors (RXRs) as master heterodimerization partners that integrate nuclear receptor signaling in the liver. They detailed isoform structure, ligands, and DNA-binding mechanisms. The authors discussed how permissive and non-permissive RXR heterodimers with PPARs, LXRs, FXR, RAR, VDR, PXR, and others orchestrate hepatic glucose, lipid, cholesterol and bile acid metabolism, as well as fibrosis and immune regulation. They critically appraised current RXR-targeting rexinoids and retinoids, highlighting metabolic and systemic toxicities. Finally, the authors argued that pathway crosstalk and genetic variation demand more selective, pharmacogenomically informed RXR modulation for future therapies for metabolic and liver diseases.

Wang et al. discussed the biology of FBXW7, the substrate-recognition component of SCF-type E3 ligases, as a central tumor suppressor frequently mutated or downregulated across gastrointestinal cancers, especially colorectal cancer, gastric cancer, esophageal squamous cell cancer, hepatocellular carcinoma, cholangiocarcinoma, and pancreatic cancer. They detailed multilayered control of FBXW7 (p53, transcription factors, miRNAs, circRNAs/lncRNAs, kinases, deubiquitinases, and dimerization) and its degradation of key oncogenic substrates, including Notch1, c-Myc, cyclin E, MCL-1, mTOR, β-catenin, and YAP. Dysfunction of this axis drives proliferation, EMT, stemness, metastasis, drug resistance, and poor prognosis. The authors outlined therapeutic concepts such as boosting FBXW7 via upstream regulators, inhibiting critical substrates (e.g., MCL-1, mTOR, EGFR), or modulating cancer stem-cell quiescence, while emphasizing challenges from pathway redundancy and *FBXW7* mutations.

Su et al. presented a Bayesian network meta-analysis of 31 randomized trials comparing six commonly used biologics (including biosimilars and combination regimens) for induction and maintenance therapy in moderate-to-severe Crohn’s disease. In TNF-antagonist-naïve patients, infliximab plus azathioprine ranked highest for induction of clinical response, while infliximab 10 mg/kg and adalimumab were most effective for maintaining remission. In patients with prior exposure to TNF antagonists, ustekinumab and risankizumab were preferred for induction therapy, with risankizumab demonstrating the most favorable safety profile in induction. Vedolizumab, including the subcutaneous formulation, did not demonstrate clear superiority in terms of efficacy or safety compared with the other biologics in the network meta-analysis.

Chen et al. reviewed gallic acid, a widely distributed plant phenolic compound, as a pleiotropic hepatoprotective agent against drug-induced liver injury and diverse xenobiotic liver insults. They summarized *in vivo* and *in vitro* evidence that gallic acid limits oxidative stress via Nrf2 activation and ROS scavenging, and suppresses inflammation through MAPK and NF-κB signaling, while modulating CYP450 activity and fibrogenic pathways. Protective effects were shown against carbon tetrachloride, antineoplastic and antituberculosis drugs, nonsteroidal anti-inflammatory drugs (NSAIDs), alcohol, biotoxins, antidepressants, and environmental toxins, with low intrinsic toxicity. The authors highlighted poor oral bioavailability and discussed phospholipid complexes, nanocarriers, network pharmacology, and liver organoids as strategies to optimize gallic acid-based interventions for drug-induced liver injury prevention and therapy.

Chaudhary et al. reviewed the signaling pathways of TGF-β superfamily ligands (TGF-βs, activins, BMPs, GDF15, inhibins, GDNF) and modulators (BAMBI) in hepatocytes. They discussed how these pathways, including canonical Smad2/3 and Smad1/5/8 pathways, as well as non-Smad cascades (TAK1-MAPK, NF-κB, PI3K-Akt, Rho GTPases), regulate hepatocyte functions such as proliferation, apoptosis, epithelial-mesenchymal transition, and hepatic stellate cell activation. These processes influence fibrosis, MASLD/metabolic dysfunction-associated steatohepatitis (MASH), and hepatocellular carcinoma (HCC), with TGF-β acting as a tumor suppressor in early stages but a tumor promoter in advanced disease. The authors also discussed the integration of TGF-β signaling with cholesterol and glucose metabolism, insulin resistance, oxidative stress, and lipotoxicity in the progression of metabolic liver disease. They highlighted potential therapies targeting this network, such as TGF-β receptor kinase inhibitors (e.g., galunisertib), dual Hedgehog/TGF-β inhibitors (e.g., Oxy210), soluble receptors, and receptor-mimicking peptides, as potential strategies for chronic liver disease and liver cancer.

Liu et al. critically appraised the review by Zeng and colleagues on anti-TNF-α biosimilars in IBD (Zeng et al., 2024). They argued that the evidence base is outdated and relies too heavily on rheumatoid arthritis data rather than IBD-specific information. The authors pointed out the lack of integration of important post-2019 studies on long-term safety, interchangeability, market penetration, and immunogenicity-guided monitoring, which reduced its relevance for current clinical decision-making. The commentary also criticized the overinterpretation of very small cohorts, the confusion between biosimilars and generics, and the incomplete discussion of regulatory and reimbursement obstacles. They called for updated, methodologically cautious syntheses focused on IBD that include real-world evidence, policy solutions, and precision-medicine tools such as trough-level and biomarker-guided switching.

Yu et al. conducted a systematic review and trial sequential analysis of 12 randomized trials, involving 1,653 elderly Chinese patients undergoing gastrointestinal endoscopy. The study compared ciprofol with propofol sedation and found that ciprofol significantly reduced hypotension, respiratory depression, hypoxemia, injection pain, involuntary movements, and nausea/vomiting. Additionally, ciprofol showed similar induction and recovery times and similar incidences of bradycardia and choking cough. The trial sequential analysis indicated that benefits for hypotension, respiratory depression, hypoxemia, and injection pain were already conclusive. The Grading of Recommendations Assessment, Development and Evaluation (GRADE) rated evidence for these key safety endpoints as moderate, but all data came from China, and the optimal ciprofol dose for endoscopy in elderly patients remains unresolved.

Chen et al. conducted a retrospective cohort study using the Medical Information Mart for Intensive Care IV (MIMIC-IV) database to investigate whether intravenous magnesium sulfate improves outcomes in intensive care unit patients with cirrhosis. Of 3,312 eligible patients, propensity-score matching resulted in 654 individuals who received magnesium treatment and 654 who did not. The study revealed that magnesium sulfate use was associated with decreased in-hospital mortality (22.0% vs. 31.0%; adjusted OR 0.47, 95% CI 0.33–0.69) and reduced 180-day mortality (adjusted HR 0.61, 95% CI 0.51–0.72), with consistent findings in sensitivity and subgroup analyses. The authors suggested potential anti-inflammatory and antioxidant mechanisms, while acknowledging the potential for residual confounding and the absence of dose and safety data, underscoring the need for further prospective validation.

Beyzaei et al. systematically reviewed 25 studies, published between 2022 and 2025, on polystyrene micro- and nanoplastics and their impact on liver health. The studies included human liver cell lines, pluripotent stem cell-derived organoids, and limited human data. *In vitro* and organoid models consistently demonstrated size- and dose-dependent oxidative stress, inflammation, apoptosis, mitochondrial dysfunction, and disruptions in lipid, sulfur amino acid, and iron balance. Nanoplastics and aged particles were identified as the most harmful. Human studies indicated the possibility of transplacental transfer, as fetal liver enzymes were elevated and microplastic burdens were higher in cirrhotic livers compared to non-diseased livers. The authors emphasized significant methodological differences and recommended standardized exposure protocols, realistic dosing, and longitudinal epidemiological studies to establish risk thresholds.

Lv et al. reported a meta-analysis of four randomized controlled trials, which included 1,807 treatment-naïve patients. The study compared vonoprazan-amoxicillin high-dose dual therapy (VPZ-HDDT) with proton pump inhibitor (PPI)-based HDDT for first-line *H. pylori* eradication. VPZ-HDDT achieved higher eradication rates than PPI-HDDT (intention-to-treat: 88.0% vs. 82.7%; per-protocol: 92.3% vs. 87.3%), with modest but statistically significant relative-risk gains. Overall adverse events (8.6% vs. 10.6%) and compliance (97.1% vs. 95.6%) were comparable between regimens. Subgroup analyses by treatment duration (10 vs. 14 days) and PPI type showed consistent trends without significant differences. All trials were conducted in China, so broader generalizability and optimal regimen duration still require confirmation in more diverse populations.

Li et al. conducted a meta-analysis of 22 randomized trials involving 7,761 patients to test opioid receptor antagonists (naloxone, naldemedine, alvimopan, methylnaltrexone, naloxegol, bevenopran) for opioid-induced constipation. These agents were found to increase weekly spontaneous bowel movements and responder rates, as well as modestly improve constipation-related quality of life, symptoms, and treatment satisfaction compared with placebo. Serious adverse events did not differ from placebo, but nonserious adverse events, mainly gastrointestinal, were more frequent and influenced by the type of drug and the duration of treatment. Most outcomes had high GRADE certainty, although some risks of bias and possible publication bias were noted, and there is still a lack of head-to-head drug comparisons.

In addition to the pharmacotherapy-focused contributions, Zhou et al. presented a systematic review and meta-analysis of the guanylate cyclase C (GCC) agonists linaclotide and plecanatide in adults with irritable bowel syndrome with constipation (IBS-C). Across nine double-blind randomized trials including 5,718 patients, linaclotide 290 μg and plecanatide 3 and 6 mg significantly increased composite responder rates, as estimated by Food and Drug Administration (FDA)-approved endpoints, versus placebo, with relative risks of 1.78, 1.63, and 1.67, respectively. These treatments consistently improved abdominal pain and complete spontaneous bowel movements, with early responses seen within 24 h. However, these benefits were accompanied by a class-related increase in diarrhea (overall RR 5.54), which was typically mild to moderate and manageable with patient counseling and dose adjustment. The PROSPERO-registered, PRISMA-compliant review applied RoB 2.0 and GRADE, providing high-certainty evidence that epithelial GCC-cGMP targeting can deliver clinically meaningful symptom relief in functional gastrointestinal disorders.

MASLD and gastrointestinal diseases represent a significant and increasing global health burden, prompting extensive research efforts aimed at better understanding their molecular basis and identifying effective therapeutic strategies. Schon and Weiskirchen reviewed chronic low-grade “silent” inflammation originating from adipose tissue as a systemic, yet often overlooked, driver of hepatic fibrogenesis in obesity and MASLD. They described how adipose-derived free fatty acids, leptin, and pro-inflammatory cytokines such as TNF-α, IL-6, and IL-1β promote hepatocyte lipotoxicity, activate Kupffer cells and hepatic stellate cells, and thereby sustain extracellular matrix deposition and progressive scarring. Beyond these mechanistic insights, the authors summarized clinical data showing that composite systemic inflammation indices (including ALI, CAR, PNI, NLR, and PLR) and classical markers such as CRP, IL-6, and procalcitonin correlate with MASLD risk, Enhanced Liver Fibrosis scores, and biomarkers of collagen formation and matrix turnover, highlighting the potential of inexpensive blood-based panels for non-invasive risk stratification. They further advocated targeting chronic low-grade inflammation through weight reduction, structured physical activity, Mediterranean-style or ketogenic dietary patterns, polyphenol-rich and microbiota-directed interventions, and other lifestyle-anchored strategies as pragmatic means to attenuate systemic inflammation and, in turn, slow or reverse the progression of liver fibrosis.

A common theme in MASLD research is mitochondrial dysfunction, which is seen as a key driver of disease progression. Deng et al. provided a comprehensive review of how impaired mitochondrial homeostasis contributes to lipid accumulation, oxidative stress, and hepatocyte injury. Their analysis highlighted altered β-oxidation, excessive reactive oxygen species production, and defective mitophagy as critical factors linking metabolic overload to cellular damage. Importantly, this work reframed mitochondria not only as energy producers but also as integrative signaling platforms whose dysfunction exacerbates endoplasmic reticulum stress, inflammatory cascades, and cell death pathways. By systematically evaluating pharmacological strategies targeting mitochondrial dynamics, redox balance, and quality control, the review emphasized mitochondrial homeostasis as a fundamental target for disease-modifying interventions in MASLD. In addition to organelle-level dysfunction, the progression of MASLD involves significant rewiring of intracellular signaling networks.

Xuan et al. focused on the Hippo-YAP1/TAZ pathway, a key regulator of cell proliferation, metabolism, and tissue regeneration. Their review elucidated how metabolic stress and lipid overload aberrantly activate YAP/TAZ signaling, promoting hepatocyte dedifferentiation, fibrosis, and tumorigenic transformation. Crucially, this work bridged metabolic disease and cancer biology, positioning Hippo-YAP/TAZ as a mechanistic link between MASLD and HCC development. The authors also discussed emerging pharmacological strategies aimed at modulating this pathway, emphasizing the therapeutic potential of targeting signaling plasticity rather than isolated metabolic enzymes.

While triglyceride accumulation has long been the focus of the narrative surrounding MASLD, there is growing evidence suggesting that cholesterol and cholesterol-associated proteins play a critical role in hepatotoxicity. In their study, Zhou et al. provided a detailed analysis of how cholesterol-binding and transport proteins contribute to liver injury by altering membrane composition, promoting oxidative stress, and amplifying inflammatory signaling. This review expanded the understanding of lipotoxicity by emphasizing that different lipid species have varying biological effects. By examining the mechanisms driven by cholesterol, the authors identified new molecular targets that could complement current lipid-lowering strategies and provide new opportunities for precision pharmacology in MASLD.

Endocrine regulation represents another critical layer in controlling metabolic diseases. Cui et al. focused on FGF21, a hepatokine with pleiotropic effects on glucose metabolism, lipid oxidation, and energy expenditure. Their review synthesized preclinical and clinical evidence supporting FGF21 analogs as promising therapeutic agents for MASLD. Notably, the authors emphasized the ability of FGF21 to restore metabolic flexibility rather than simply suppress lipid accumulation. MASLD does not develop in isolation from the gastrointestinal system.

Li et al. systematically reviewed randomized controlled trials of PPAR agonists in non-alcoholic fatty liver disease (NAFLD/MASLD), covering subtype-selective, dual, and pan-PPAR agonists across the spectrum from simple steatosis to biopsy-proven steatohepatitis. The authors highlighted that PPARα and PPARβ/δ agonists such as fenofibrate, pemafibrate, and seladelpar robustly improve circulating triglycerides and other atherogenic lipids. However, to date, they have shown only modest effects on intrahepatic fat content and histological endpoint. In contrast, the PPARγ agonist pioglitazone is consistently associated with clinically meaningful improvements in steatohepatitis activity, insulin resistance, and liver fat, particularly in patients with NASH and coexisting type 2 diabetes or prediabetes, although its broader use is curtailed by predictable adverse effects, including weight gain, edema, and increased fracture risk. The authors further discussed next-generation PPARγ-modulating agents (such as MSDC-0602K and PXL065), dual PPARα/γ agonists like saroglitazar, and pan-PPAR agonists exemplified by lanifibranor, which together illustrate how more selective modulation, rational combination therapy, and biomarker-guided patient stratification could better harness PPAR signaling as a disease-modifying strategy in MASLD.

Shichkin reviewed the combined use of vitamin B5 and vitamin U in mucosa-associated gastrointestinal pathologies, highlighting their roles in epithelial integrity, antioxidant defense, and metabolic support. Although the review was centered on gastrointestinal tissues, this work has important implications for MASLD, given the pivotal role of gut barrier function and nutrient absorption in shaping hepatic metabolic load.

Complementing this perspective, Vari et al. explored the pharmacological potential of endocannabinoid and endocannabinoid-like compounds in preserving intestinal structure and metabolism under high-fat conditions. Their review underscored the gut-liver axis as a therapeutic target, demonstrating how modulation of intestinal homeostasis can indirectly alleviate hepatic metabolic stress and inflammation.

HCC represents a frequent endpoint of chronic metabolic liver disease. Liu et al. contributed original research evaluating the efficacy and safety of combining transarterial chemoembolization with hepatic arterial infusion chemotherapy, tyrosine kinase inhibitors, and immune checkpoint inhibitors in advanced HCC. This study exemplifies the translational relevance of understanding metabolic disease mechanisms, as metabolic dysfunction profoundly influences tumor biology, treatment response, and the immune microenvironment. The integration of locoregional and systemic therapies reflects the necessity of multimodal approaches in metabolically driven cancers.

In conclusion, the articles in this issue demonstrate how mechanistic insight and rigorous clinical research can work together to reshape the management of gastrointestinal and liver diseases. By clarifying pathogenic pathways, improving existing therapies, and critically evaluating emerging interventions, they point toward more targeted, safer, and truly patient-centered care.

## References

[B1] Devarbhavi H. Asrani S. K. Arab J. P. Nartey Y. A. Pose E. Kamath P. S. (2023). Global burden of liver disease: 2023 update. J. Hepatol. 79 (2), 516–537. 10.1016/j.jhep.2023.03.017 36990226

[B2] Gan C. Yuan Y. Shen H. Gao J. Kong X. Che Z. (2025). Liver diseases: epidemiology, causes, trends and predictions. Signal Transduct. Target Ther. 10 (1), 33. 10.1038/s41392-024-02072-z 39904973 PMC11794951

[B3] Zeng Z. Lin H. Jiang M. Yuan J. Li X. Jia Y. (2024). Anti-TNFα in inflammatory bowel disease: from originators to biosimilars. Front. Pharmacol. 15, 1424606. 10.3389/fphar.2024.1424606 39114362 PMC11303209

